# Association of NCOA6 Gene Polymorphism with Milk Production Traits in Chinese Holstein Cows

**DOI:** 10.3390/ani15101461

**Published:** 2025-05-19

**Authors:** Muhammad Talha Bin Tahir, Sahar Ghulam Mohyuddin, Yiyang Yao, Yanru Wang, Yan Liang, Niel A. Karrow, Yongjiang Mao

**Affiliations:** 1 Key Laboratory for Animal Genetics, Breeding, Reproduction and Molecular Design of Jiangsu Province, College of Animal Science and Technology, Yangzhou University, Yangzhou 225009, China; talhatahir2640@gmail.com (M.T.B.T.); saharbutt-18@hotmail.com (S.G.M.); dx120240186@stu.yzu.edu.cn (Y.Y.); wyr0821wyr@163.com (Y.W.); mz120181016@yzu.edu.cn (Y.L.); 2 Joint International Research Laboratory of Agriculture and Agri-Product Safety of the Ministry of Education of China, Yangzhou University, Yangzhou 225009, China; 3 Center for Genetic Improvement of Livestock, Department of Animal Biosciences, University of Guelph, Guelph, ON N1G2W1, Canada; nkarrow2@gmail.com

**Keywords:** Holstein cows, SNPs, NCOA6, milk production traits

## Abstract

Nuclear receptor coactivator 6 is a gene that produces a protein that regulates transcriptional activity. It is also involved in many processes like cell survival, metabolism, homeostasis, and embryonic development. This study focused on studying the genetic influence of single-nucleotide polymorphisms (SNPs) within the NCOA6 gene on lactation performance characteristics. The objective of this study was to determine the genetic impact of SNPs of the NCOA6 gene on milk production traits. After the random selection of twenty cows, PCR amplification and sequencing were performed from their blood samples to find the SNPs present in the bovine NCOA6 gene. As a result, two SNPs, g.71544C > T and g.87310A > G, were found. Then, the genotyping of the cows (*n* = 985) was conducted using a Sequenom MassARRAY based on previously identified SNP information. The least-square method was applied to study the links between lactation traits, somatic cell score (SSC), and 305-day milk output. The results indicated that the SNP g.87310A > G was strongly associated with g.71544C > T in linkage disequilibrium. The single-nucleotide polymorphism g.87310A > G showed a very strong association with daily milk yield and 305-day milk production. Individuals with the AA genotype exhibited a notable increase in daily milk production by 1.64 kg, and their 305-day milk yield was approximately 500 kg higher. This variation demonstrated a significant genetic effect on milk output. In brief, the pleiotropic influence of the cattle NCOA6 gene on lactation traits was revealed in this study. However, additional research will provide a basis for further exploring the underlying correlation and theoretical framework of the molecular genetics of milk composition and the production attributes of Holstein cows.

## Simple Summary

Milk is the most vital commodity that is used worldwide. In the dairy industry, traits that are linked with milk production are of economic importance, increasing the focus of dairy farmers on enhancing milk quality. Nuclear receptor coactivator 6 is a gene that is involved in controlling gene expression by encoding a protein that enhances the activity of transcription factors. It is involved in many cellular and homeostatic processes. An SNP is a variation in a single nucleotide at a specific position in a DNA location. These polymorphisms affect the milk production traits of dairy cows. In this study, we identified two SNPs, g.71544C > T and g.87310A > G, in the NCOA6 gene in Chinese Holstein cows, which ultimately affected the 305-day milk yield, fat content, and to some extent the somatic cell score. The reference sequences of these two SNPs were taken from the NCOA6 Bos taurus gene on chromosome 13, having a reference sequence number of NC_037340.1. In brief, polymorphisms of the NCOA6 gene for milk production traits were studied, serving as a valuable reference in the genetic selection of Chinese Holstein cows and supporting improved economic returns.

## 1. Introduction

Holsteins are a world-famous breed of dairy cows worldwide, known for their high milk production capacity [1]. The cows of this breed are typically characterized by their distinctive white-and-black appearance, and Holsteins are the largest and most widespread dairy breed globally [2]. They are found in almost one hundred and fifty countries, with a total population of about seventy million head of cattle [3].

Dairy management is an economically important aspect of screening daily milk output. Numerous genetic, physiological, and environmental factors influence the milk production capacity of dairy cows; however, certain critical elements directly impact milk yield as well as production capability [4]. In dairy farming, lactation traits are of great importance. These traits are created through breeding programs. In breeding, selection is one of the most important factors. Various traditional selection methods are used, but genomic selection enhances the effectiveness of selection. DNA-based selection methods focus on a quantitative trait through identifying the key genes that regulate traits that are of economic importance [5,6].

There is a significant correlation among milk traits, such as fat content (FC), milk yield, protein content (PC), milk urea nitrogen (MUN), and somatic cell count (SSC) [7]. Furthermore, mastitis, which is characterized by udder inflammation, is the most widespread ailment in milk-producing cows and results in drops in milk production and milk quality [8]. The milk somatic cell score is considered an indirect selection strategy that decreases the risk of clinical mastitis, with which it is positively correlated [9]. Recently, major improvements have been achieved in the physiological aspects of milk production of Holstein cows [10]. The current dairy industry has transformed through advances in the genomic information available and analytical techniques, which have opened a new window of opportunity to find certain genomic regions related to complex phenotypic traits [11,12]. Recent studies revealed that identifying the single-nucleotide polymorphisms (SNPs) and genes linked to dairy output during the 305 d lactation period may assist in discovering the genes that are associated with milk production traits in dairy cows [13]. An SNP is defined as change in the DNA sequence where a single nucleotide differs from the normal sequence, for instance, changing nucleotides in specific DNA locations, such as replacing cytosine (C) with adenine (A) [14]. The nuclear receptor coactivator (NCOA6) is an important and multifunctional coactivator for certain transcriptional factors and nuclear hormone receptors [15]. This gene is found in many tissues like the brain, ovary, testis, fat, heart, and liver [16] as well as in in the mammary glands of mice [17]. *NCOA6* is involved in many biological processes like growth, energy metabolism, cell survival, and wound healing [18]; it is also vital for embryonic development [17] and plays a main role in the development of mammary glands [19]. Distinct isoforms of *NCOA6* have been identified in the mammary glands of mice at various stages of developmental such as in adult virgins as well as during pregnancy, lactation, and involution [16]. In dairy cows, the nuclear receptor *NCOA6* interacts with PPARγ, a transcription factor, thereby influencing the regulation of milk fat synthesis [15]. PPARγ influences the expression of genes engaged in the transport of fatty acid, such as ACSL1, LPL, and CD36 [20], and was found to be as a prominent controller in the synthesis of fat in of bovine milk [21]. A study identified NCOA6, also known as the peroxisome proliferator-activated receptor-interacting protein (PRIP) as a key factor involved in PPARα/RXRα signaling within the gene regulatory networks active during lactation [22].

In this study, we sequenced the target exonic areas of the NCOA6 gene in Bos taurus, identified on chromosome 13. However, to the best of our knowledge, there are no studies specifically validating the role of the NCOA6 gene in daily milk production and 305d milk production. Therefore, we hypothesized that SNPs in the NCOA6 gene contribute to the variation in daily and 305d milk production. Thus, this study aimed to examine the potential interconnection of NCOA6 with dairy yield characteristics among Holstein cattle in Southern China.

## 2. Materials and Methods

All animal welfare protocols were strictly obeyed during these experiments, following the guidelines set forth by the Institutional Administrative Committee and the Ethics Committee of Laboratory Animals (license number: SYXK [Su] 2017-0044), as issued by the Yangzhou University Institutional Animal Care and Use Committee. The phenotypic data were collected from 985 Holstein cows, comprising 9076 test day records, across various cattle herds located in China (Jiangsu Province). All animals were housed in tie-free stalls and provided with a Total Mixed Ration (TMR) on a dry matter (DM) basis mentioned in Table 1**.**

### 2.1. Sampling and Data Collection

Data were collected using the Dairy Cow Management DC-305 software (Valley Ag. Software, San Francisco, CA, USA). The dataset was categorized considering the following parameters: Test Day Milk Yield (TDMY), ranging from 5 to 60 kg; fat content (FC), ranging from 2% to 7%; protein content (PC), ranging from 2% to 6%; somatic cell score (SCS), ranging from 0 to 9; and 305-day milk yield. These classifications ensured data uniformity and reliability for subsequent statistical analysis. In this study, 9076 test day records were reviewed. Blood samples from 985 Chinese Holstein cows were sampled across six different herds in Jiangsu province, China. About 6 mL of blood was taken from the caudal vein of each cow and stored under appropriate temperature conditions, i.e., −80 °C.

### 2.2. DNA Isolation and SNP Profiling

The traditional DNA extraction method was applied to isolate DNA from the blood samples collected from each cow’s tail vein. The phenol–chloroform DNA extraction protocol was followed, and DNA was dissolved in TE buffer. Tris-EDTA buffer serves as a diffusing agent, protecting nucleic acids from enzyme degradation [23]. Certain samples of DNA were diluted to a concentration of 100 g µL^−1^ and stored at −20 °C for future use.

The optimal binding temperature was determined to establish the polymerase chain reaction temperature gradient **(**Table 2), and the PCR amplification was performed in a PTC-200 DNA Engine cycler (Bio-Rad, Big Sur, CA, USA). To identify the SNP sites and their precise region, 20 bovine DNA samples were randomly picked from a total pool of 985 samples. Sequencing polymerase chain reaction (PCR) was employed to detect all the SNPs in the bovine *NCOA6* gene. The amplification products were confirmed through agarose gel electrophoresis, and the results were verified by sequencing performed by Sangon Biotech (Shanghai, China).

### 2.3. DNA Sequencing, Amplification, and SNP Genotyping of Individual Samples

According to the NCBI Gene Database, thirteen primers were designed, and the effectiveness of amplification was verified through agarose gel electrophoresis. Sequencing was subsequently carried out by Sangon Biotech (Shanghai, China). The primer sequences and sites of mutation were studied, and the locations of primers were identified using 3 bioinformatics software tools: SeqMan version 12.3 (Invitrogen, Carlsbad, CA, USA), SnapGene Viewer version 7.0.3 (Invitrogen, Carlsbad, CA, USA), and Vector NTI version 11.5.4 (Invitrogen, Carlsbad, CA, USA). Following the identification of SNP sites, MassARRAY technology (Sequenom Inc., San Diego, CA, USA) was utilized for genotyping all 985 samples, including the initial 20 samples. To confirm the accuracy of SNP analysis, 20 samples were analyzed in duplicate sets (the tester was unaware of the fact that these samples had been duplicated). The study showed one hundred percent accuracy in SNP genotyping.

### 2.4. Statistical Analysis

Sequencing data for each individual were used to identify SNP regions, and the allelic and genotypic frequencies were calculated for each point. The Hardy–Weinberg equilibrium (HWE) for each polymorphism was tested by applying a statistical test (chi-square). SHEsis (http://analysis.bio-x.cn/SHEsisMain.htm (accessed on 5 October 2023)) focused on conventional genetic analytical methods (containing genotype frequency, HWE, gene frequency, and linkage disequilibrium analysis among other factors [24,25]. The haplotype analysis of each cow was conducted using Beagle 5.1 software (Brian L. Browning, Washington, DC, USA) [26]. The relationships between milk production characteristics and somatic cell score (SCS) were reviewed. Due to the inadequate distribution of the SCC in statistical testing, it was transformed to SCS using the formula SCS = log2 (SCC/100) + 3, and genotypes and haplotypes were evaluated using the least-square approach and General Linear Model (GLM) from SPSS Ver26.0 (IBM, Armonk, New York, NY, USA) [26,27]. The model was as follows:

Y*_ijklmnopq_* = µ + Year*_i_* + Season*_j_* + Parity*_k_* + CS*_l_* + DIM*_m_* + F*_n_* + G*_o_* + e*_ijklmnop_* (1)

In the above equation, Y*_ijklmnop_* is the dependent variable, which includes traits such as total daily milk yield (TDMY), fat content (FC), protein content (PC), somatic cell score (SCS), and lactation yield. The term μ represents the overall mean; Year*_i_* represents the set impact of the *i*-th year (where *i* is 2016–2018); Season*_j_* is a set impact of the *j*th test season, which includes spring (March to May), summer (June to August), fall (September to November), winter (December to January), and February of the next year; Parity*_k_* represents the set impact of the *k*th parity, where cows’ parity ranges from 1 to 3; CS*_l_* is the set impact of the *l*th calving period, where the calving period line up according to the corresponding test period divisions; DIM*_m_* is the set impact of the *m*th days in milk (DIM), categorized into three levels: <100 days, 100–200 days, and >200 days; F*_n_* is the set effect of the *n*th farm, with *n* is equal to 6, demonstrating distinct dairy farms located in six various regions of Jiangsu Province, China; G*_o_* is the set impact of the *o*th genotype or haplotype; and e*_ijklmnop_* is the arbitrary residual error term. Statistical significance was established at *p* < 0.05. Duncan’s multiple range test was used to conduct multiple comparisons among the various degrees of these variables.

## 3. Results

### 3.1. SNP Analysis of the Bovine NCOA6 Gene: Genotypic and Allelic Frequencies with Evaluation of the Hardy–Weinberg Equilibrium

This study analyzed the collected DNA of twenty randomly selected Chinese Holstein cows and identified the site of two novel SNPs in the NCOA6 gene (Figure S1) in the Supplementary File. g71544C > T and g87310A > G were positioned in the exonic coding region in the 5′ to 3′ direction. The genotypic and allelic frequencies of two SNP loci in the NCOA6 gene were determined using the chi-square test and the Hardy–Weinberg equilibrium equation (Table 3). The chi-square value in the Hardy–Weinberg equilibrium for two SNPs is shown in Table 3. The total set of animals carrying two particular SNPs was 908 and 974 for g.71544C> T and g.87310A > G, respectively (Table 3).

### 3.2. Haplotype Analysis of the NCOA6 Gene in Chinese Holstein Cows

Haplotype analysis was performed by using the Haploview tool Beagle 5.1 (Brian L. Browning, Washington, DC, USA). The results showed no linkage disequilibrium among the SNPs, indicating a low level of genetic association within the analyzed blocks. The value of r^2^ was 0.046 between g87310A > G and g71544C > T. Three haplotypes were constructed as CA, CG, and TA, having frequencies of 0.525, 0.413, and 0.061, respectively.

### 3.3. Impact of Various Non-Genetic Factors on Milking Characteristics, 305-Day Milk Yield, and Somatic Cell Score (SCS)

Table 4 presents the effects of various non-genomic factors on lactation traits, 305-day milk yield, and somatic cell score (SCS). The cattle farm, parity, year, and calving season showed a strong, statistically significant effect on 305 d milk output (*p* < 0.01), while the cattle farm and parity showed a significantly marked effect on somatic cell score (*p* < 0.01). Lactation days showed a highly significant impact on milk production and milk fat content (*p* < 0.01). Meanwhile, the testing season has a marked influence on protein percentage (*p* < 0.05).

### 3.4. Connection of SNPs in the NCOA6 Gene with Lactation Performance and Somatic Cell Score (SCS) in Holstein Cattle

Based on the results, SNP g87310A > G was almost totally linked. Furthermore, we analyzed the effect of g71544C > T on somatic cell score (SCS) and lactation traits. The observed outcomes of the *NCOA6* gene on lactation traits and somatic cell score (SCS) are presented in Table 5. The *NCOA6*-87310A > G SNP site was linked significantly with milk output, fat content, and 305-day milk production. However, there was no association of NCOA6-71544C > T with any measured traits.

The SNP NCOA6-87310A > G had a statistically significant impact on fat percentage and TDMY (*p* < 0.05) and a highly notable impact on 305-day milk production (*p* <0.01). Cows with the AA genotype had a significantly higher test-day milk yield than those with the GG genotype (*p* <0.05). However, GA and GG genotypes had no statistical difference. Similarly, the milk fat percentage of the GG genotype was more statistically significant than the GA genotype (*p* < 0.05), and the GG genotype of 305-day milk production was more important as compared to the AA and GA genotypes (*p* <0.05).

## 4. Discussion

Due to improved economic levels and greater awareness of health risks and healthy diets, milk consumption has also increased in recent times. The factors that mark traits of milk production, such as 305d milk output, fat percentage, fat yield, protein percentage, and yield, are also increasingly receiving attention. The increase in milk yield represents the economic value, while the percentage changes are related to milk quality. To improve milk production traits controlled by multiple genes with micro-effects, individuals with specific genotypes of related genes can be screened through molecular breeding techniques. Since 2009, genomic selection (GS) has been successively used in bovine breeding in many countries. This approach uses single-nucleotide polymorphism (SNP) markers to enhance selection effectiveness and genetic precision [28]. Cattle breeding has been revolutionized through genomic selection. It enables prediction of genetic merit by using the single-nucleotide polymorphism as a marker. The accuracy of breeding value estimation has greatly increased through this method, especially for complicated traits like milk output. SNP markers have improved selection accuracy and genetic gain by enabling the more accurate identification of genetic variants linked to fat content, protein content, and milk yield in breeding operations [29,30,31]. As SNPs in candidate functional genes with significant genetic effects can affect milk production traits, adding wide range of genomic markers with relevant information to expand SNP marker data can improve the precision of estimated genomic breeding value prediction [32]. Nuclear receptor coactivator 6 (*NCOA6*) is a vital coactivator that interacts with certain transcription factors and nuclear hormone receptors [16]. It is revealed in different tissues, including the liver, brain, testis, fat, ovary, heart, and mammary gland [16,17]. Olsen etc. [15] found that *NCOA6* is a nuclear receptor coactivator that associates with transcription factors like PPARγ, a crucial regulator of milk fat synthesis in cattle. Despite its potential relevance, limited information is present regarding the role of *NCOA6* in milk output. In this study, we identified two novel SNPs in the *NCOA6* gene in Holstein cattle: g.87310A > G and g.71544C > T. These SNPs were significantly associated with lactation performance traits. According to our findings, this study is the first to explore the association among SNPs in the *NCOA6* gene and lactation traits in Holstein cows. Desvergne [20] reported that downregulation of the *NCOA6* gene increases milk production through the binding of transcription factors to the peroxisome proliferator–activator receptor. As a result, a depression in milk fat synthesis takes place. This study showed that there is a significant association of SNPs with milk output and milk production traits in the *NCOA6* gene. The SNP g.87310A > G showed significant associations with 305d milk production and milk fat contents. *NCOA6* is identified as a crucial component in PPARα/RXRα signaling in a study examining the regulatory networks of genes involved in lactation, where *NCOA6* is referred to as PRIP [22]. Thus, *NCOA6* may operate as both an operational and functional potential gene for the quantitative trait loci (QTL) situated on BTA13 [15]. From the above studies, it is obvious that SNPs present in the *NCOA6* gene significantly influence the lactation traits of Holstein cattle. However, there is a greater need for research on this gene to better understand more SNPs and correlate their effect with milk production traits. The presented results support the previously mentioned hypothesis that *NCOA6* significantly influences both daily and 305-day milk production. Research indicates that polymorphisms in the *NCOA6* gene greatly affect the lactation efficiency of Holstein cows. These findings underscore the significance of *NCOA6* as a pivotal genetic regulator affecting milk production parameters, suggesting that further investigation into this gene could enhance our understanding of its role in bovine breeding.

## 5. Conclusions

In conclusion, the 87310 A > G variant of the *NCOA6* gene was screened through SNP screening technology, and it was found that the mutation was highly correlated to daily milk yield and 305-day milk yield of dairy cows, the daily milk yield of AA-type individuals was significantly increased by 1.64 kg, and the milk yield of 305 days was significantly increased by about 500 kg, which had a significant genetic effect. Further investigation is necessary to assess the impact of these SNPs on functional and practical applications for enhanced breeding methods. In future, large-scale, meticulously organized investigations should be conducted, as these SNPs possess the capacity to alter gene expression.

## Figures and Tables

**Table 1:** Total Mixed Ration (TMR) nutrient components and composition based on dry matter.

Component Ingredient	Composition (%)
Lucerne hay	20.00
Silage (corn)	25.00
Wheat straw	8.00
Barley grain	15.50
Canola meal	7.00
Sunflower meal	5.00
Beet pulp	4.50
Soya hulls	5.50
Limestone	0.40
Sodium bicarbonate (NaHCO_3_)	0.35
Salt (NaCl)	0.30
Magnesium oxide (MgO)	0.20
Vitamin mineral premix	1.25

The vitamin–mineral premix provided the following per kg of concentrate: 300,000 IU of vitamin A, 450,000 IU of vitamin D, 1400 IU of vitamin E, 550 mg of nicotinic acid, 700 mg of Cu, 900 mg of Mn, 1200 mg of Fe, 3500 mg of Zn, 20 mg of Se, 12 mg of Co, and 8 mg of I. The Net Energy for Lactation is 1.42 Mcal/kg, calculated using updated feed evaluation systems according to NRC, 2021.

**Table 2:** The primer sequences, product size, and annealing temperature used for PCR amplification of the *NCOA6* gene.

Primer	(5′-3′) Primer Sequences	Production Size (Base Pair)	Position	Exon	Annealing Temperature (°C)
P1	F: TAGTTATGTTCTTCTTGTGCTTC R: TTATTCAGTTCTACTTCCAACAC	649	53,773–54,421	5′UTR +Exon 1	50.5
P2	F: ATGAGATTGGGAAGGAGTAGGAG R: AATTCTTGGTTATGGAGGAGCAG	608	61,210–61,817	Exon 2 + Intron 2	58
P3	F: TGCTCACAAACATTAAATGATACC R: CATTTCACTCCCTCCTTTAACTC	1080	62,143–63,222	Exon 3 + Intron 3	55.6
P4	F: CCTCAGATCACATTTAGGGAGCAG R: CGTCTATGGCTACTTTGGTGCTAC	765	64,895–65,659	Exon 4 + Intron 4	53
P5	F: ACATCTGTTTATGCTGATCACTGG R: GATTTCAGACAAGACTATCACAACC	1274	67,749–69,022	Exon 5 + Intron 5	55.2
P6	F: GGTTGAATTTGATAGCTACCC R: CTGACAAGAATAAAGAGGCAC	798	71,207–72,004	Exon 6 + Intron 6	48.6
P7	F: GATTTTGGGGGCTCGTTTTAG R: AGAATCTAATTTGTGAGTCTGTGGG	1013	75,206–76,218	Exon 7,1 + Intron 7,1	56.7
P8	F: CCAGAAAGACTCAATGCCTCC R: GTCCCTCAGAAACCATAACCTTG	1210	76,015–77,224	Exon 7,2 + Intron 7,2	54.7
P9	F: CCAATCCCATCACAACTTCAG R: CTTCCTCCAAGTAGAAAAGGAG	1177	77,102–78,278	Exon 7,3 + Intron 7,3	54.7
P10	F: CTGAAAGAGGTTTGGGTTGCC R: GAGATGCCCTTCTTGAGTTCC	664	80,748–81,411	Exon 8 + Intron 8	52.4
P11	F: ACGGGATTATTTCACAGTATGG R: AGTGAGGTCGAAGCTACAGTTG	764	83,185–83,948	Exon 9 + Intron 9	52.2
P12	F: GTATTGGTTCTGCCATGTATC R: CTAAGCAGCAGAAGTCAAAGC	604	86,966–87,569	Exon 10 + Intron 10	52.3
P13	F: CAGTCACTCGCTTGTAGCATC R: GATTCTCTTTATTCACTGGTCC	904	95,425–96,328	Exon 11 + 3′ UTR	51.4

**Table 3:** Genotypic, allelic frequency, and HWE test of two mutations in the *NCOA6* gene.

SNP Locus	Genotype	Genotype Frequency	Sample Number	Allele	Allele Frequency	H-W Value	Pearson’s *p*-Value
*NCOA6*-71544C > T	CC	0.87	798	C	0.93	0.69	0.40
	CT	0.87	108	T	0.06		
	TT	0.00	2				
*NCOA6*-87310A > G	AA	0.35	342	A	0.58	1.41	0.23
	AG	0.46	455	G	0.41		
	GG	0.18	177				

HWE stands for Hardy–Weinberg equilibrium.

**Table 4:** Influence of diverse non-genetic factors on lactation traits, somatic cell score (SCS), and 305-day milk production.

Factor	Milking Trait and SCS	F-Value	Sig.
Cow farm	Milk yield (kg)	9.032 **	0.000
	Milk fat content (%)	7.083 **	0.000
	Protein content (%)	16.277 **	0.000
	Somatic cell fraction	14.452 **	0.000
	305-day milk yield (kg)	15.858 **	0.000
Parity	Milk yield (kg)	1.938	0.144
	Milk fat content (%)	1.125	0.325
	Protein content (%)	3.098	0.045
	Somatic cell fraction	10.188 **	0.000
	305-day milk yield (kg)	12.478 **	0.000
Test year	Milk yield (kg)	4.201 *	0.015
	Milk fat content (%)	0.390	0.677
	Protein content (%)	1.088	0.337
	Somatic cell fraction	0.573	0.564
	305-day milk yield (kg)	29.506 **	0.000
Calving season	Milk yield (kg)	2.918	0.033
	Milk fat content (%)	0.644	0.587
	Protein content (%)	0.594	0.619
	Somatic cell fraction	3.380	0.017
	305-day milk yield (kg)	8.295 **	0.000
Days in milk	Milk yield (kg)	34.020 **	0.000
	Milk fat content (%)	8.603 **	0.000
	Protein content (%)	48.805	0.000
	Somatic cell fraction	1.245	0.292
	305-day milk yield (kg)	1.176	0.317
Testing season	Milk yield (kg)	2.183	0.088
	Milk fat content (%)	1.470	0.221
	Protein content (%)	5.744 **	0.001
	Somatic cell fraction	0.196	0.899
	305-day milk yield (kg)	1.530	0.205

The analysis of variance (ANOVA) employs a joint hypothesis testing approach using the F-test. The F-value obtained from the test formula indicates a particular value, which is then used to find the corresponding *p*-value through tables or alternative methods, representing the significance level (Sig). The asterisks represent statistical significance; * denotes *p* < 0.05, and ** denotes *p* < 0.01.

**Table 5:** Effects of different genotypes based on the *NCOA6* gene on lactating performance and SCS in Holstein cattle.

SNP Locus	Genotype	Number of Records	TDMY (kg)	Fat (%)	Protein in Milk (%)	Somatic Cell Score	305-Day Milk Production
*NCOA6*-71544C > T	CC	7488	35.207 ± 0.127	3.635 ± 0.010	3.229 ± 0.004	2.77 ± 0.05	10,220.45 ± 28.577
	CT	1032	34.983 ± 0.351	3.569 ± 0.028	3.192 ± 0.011	2.86 ± 0.0	10,366.01 ± 97.404
	TT	4	41.000 ± 5.354	3.766 ± 0.582	3.046 ± 0.078	2.75 ± 0.854	11,339.00 ± 424.000
	Total	8524	34.965 ± 0.019	3.643 ± 0.010	3.235 ± 0.004	2.76 ± 0.02	10,189.26 ± 27.745
	F-value						
	Sig						
*NCOA6*-87310A > G	AA	3207	35.289 ± 0.194 ^a^	3.650 ± 0.016 ^ab^	3.230 ± 0.006	2.80 ± 0.037	10,451.31 ± 46.781 ^a^
	GA	4230	34.915 ± 0.167 ^ab^	3.621 ± 0.013 ^b^	3.236 ± 0.006	2.68 ± 0.032	10,101.19 ± 38.360 ^b^
	GG	1588	34.605 ± 0.273 ^b^	3.685 ± 0.023 ^a^	3.241 ± 0.009	2.90 ± 0.054	9947.85 ± 60.642 ^c^
	Total	9025	35.181 ± 0.115	3.657 ± 0.010	3.224 ± 0.004	2.78 ± 0.022	10,238.448 ± 26.840
	F-value		4.209 *	3.247 *	1.404	2.713	22.886 **
	Sig		0.015	0.039	0.246	0.066	0.000

The analysis of variance (ANOVA) utilizes the F-test method. The F-value obtained from the test formula denotes a particular value used to determine the corresponding *p*-value through lookup tables or other techniques, indicating statistical significance (Sig). The * (*p* < 0.05) and ** (*p* < 0.01) values marked with a, b, and c within the same column indicate significant differences at *p* < 0.05.

## Data Availability

The original contributions presented in this study are included in the article. Further inquiries can be directed to the corresponding author.

## Supplementary Materials

The following supporting information can be downloaded at: https://www.mdpi.com/article/10.3390/ani15101461/s1, Figure S1: Nuclear receptor coactivator 6 gene with the localization of one identified SNP (NCOA6).

## References

[1] Barua K., Akter N., Alam M., Bari M.S., Sultan M.N., Islam S., Hossain M.E. (2022). Effects of Genotype, Parity, Season and Their Interactions on Milk Yield in Crossbred Dairy Cattle. J. Anim. Physiol. Anim. Nutr..

[2] Ozdemir M., Motmain Z., Ekinci K., Saygılı E. (2024). Associations Between BLG, CSN3, DGAT1, GH, PIT1, and PRL Gene Polymorphisms and Milk Production Traits in Holstein Dairy Cows: A Meta-Analysis. Biochem. Genet..

[3] Andrew W., Gao J., Mullan S., Campbell N., Dowsey A.W., Burghardt T. (2021). Visual Identification of Individual Holstein-Friesian Cattle via Deep Metric Learning. Comput. Electron. Agric..

[4] Oliveira H.R., Cant J.P., Brito L.F., Feitosa F.L.B., Chud T.C.S., Fonseca P.A.S., Jamrozik J., Silva F.F., Lourenco D.A.L., Schenkel F.S. (2019). Genome-Wide Association for Milk Production Traits and Somatic Cell Score in Different Lactation Stages of Ayrshire, Holstein, and Jersey Dairy Cattle. J. Dairy Sci..

[5] Anggraeni A., Sumantri C., Saputra F., Praharani L. (2020). Association between GH (g.1456_1457insT), GHRH (g.4474 C>a), and Pit-1 (g.244G>A) Polymorphisms and Lactation Traits in Holstein Friesian Cattle. Trop. Anim. Sci. J..

[6] Thuy N.T.D., Thu N.T., Cuong N.H., Ty L.V., Nguyen T.T.B., Khoa D.V.A. (2018). Polymorphism of PIT-1 and Prolactin Genes and Their Effects on Milk Yield in Holstein Frisian Dairy Cows Bred in Vietnam. Russ. J. Genet..

[7] Jattawa D., Koonawootrittriron S., Elzo M.A., Suwanasopee T. (2012). Somatic Cells Count and Its Genetic Association with Milk Yield in Dairy Cattle Raised under Thai Tropical Environmental Conditions. Asian Australas. J. Anim. Sci..

[8] Gussmann M., Steeneveld W., Kirkeby C., Hogeveen H., Farre M., Halasa T. (2019). Economic and Epidemiological Impact of Different Intervention Strategies for Subclinical and Clinical Mastitis. Prev. Vet. Med..

[9] Rupp R., Boichard D. (1999). Genetic Parameters for Clinical Mastitis, Somatic Cell Score, Production, Udder Type Traits, and Milking Ease in First Lactation Holsteins. J. Dairy Sci..

[10] Akers R.M. (2017). A 100-Year Review: Mammary Development and Lactation. J. Dairy Sci..

[11] Miglior F., Fleming A., Malchiodi F., Brito L.F., Martin P., Baes C.F. (2017). A 100-Year Review: Identification and Genetic Selection of Economically Important Traits in Dairy Cattle. J. Dairy Sci..

[12] Bobbo T., Tiezzi F., Penasa M., De Marchi M., Cassandro M. (2018). Short Communication: Association Analysis of Diacylglycerol Acyltransferase (DGAT1) Mutation on Chromosome 14 for Milk Yield and Composition Traits, Somatic Cell Score, and Coagulation Properties in Holstein Bulls. J. Dairy Sci..

[13] Clancey E., Kiser J.N., Moraes J.G.N., Dalton J.C., Spencer T.E., Neibergs H.L. (2019). Genome-Wide Association Analysis and Gene Set Enrichment Analysis with SNP Data Identify Genes Associated with 305-Day Milk Yield in Holstein Dairy Cows. Anim. Genet..

[14] Brookes A.J. (2005). Single Nucleotide Polymorphism (SNP). Encycl. Life Sci..

[15] Olsen H.G., Knutsen T.M., Kohler A., Svendsen M., Gidskehaug L., Grove H., Nome T., Sodeland M., Sundsaasen K.K., Kent M.P. (2017). Genome-Wide Association Mapping for Milk Fat Composition and Fine Mapping of a QTL for de Novo Synthesis of Milk Fatty Acids on Bovine Chromosome 13. Genet. Sel. Evol..

[16] Li Q., Xu J. (2011). Identification and Characterization of the Alternatively Spliced Nuclear Receptor Coactivator-6 Isoforms. Int. J. Biol. Sci..

[17] Lemay D.G., Lynn D.J., Martin W.F., Neville M.C., Casey T.M., Rincon G., Kriventseva E.V., Barris W.C., Hinrichs A.S., Molenaar A.J. (2009). The Bovine Lactation Genome: Insights into the Evolution of Mammalian Milk. Genome Biol..

[18] Mahajan M.A., Samuels H.H. (2008). Nuclear Receptor Coactivator/Coregulator NCoA6(NRC) Is a Pleiotropic Coregulator Involved in Transcription, Cell Survival, Growth and Development. Nucl. Recept. Signal..

[19] Qi C., Kashireddy P., Zhu Y.T., Rao S.M., Zhu Y.J. (2004). Null Mutation of Peroxisome Proliferator-Activated Receptor-Interacting Protein in Mammary Glands Causes Defective Mammopoiesis. J. Biol. Chem..

[20] Desvergne B., Michalik L., Wahli W. (2006). Transcriptional Regulation of Metabolism. Physiol. Rev..

[21] Bionaz M., Loor J.J. (2008). Gene Networks Driving Bovine Milk Fat Synthesis during the Lactation Cycle. BMC Genomics.

[22] Lemay D.G., Neville M.C., Rudolph M.C., Pollard K.S., German J.B. (2007). Gene Regulatory Networks in Lactation: Identification of Global Principles Using Bioinformatics. BMC Syst. Biol..

[23] Liang Y., Gao Q., Zhang Q., Adam A., Arbab I., Li M., Yang Z., Karrow N.A., Mao Y. (2020). Polymorphisms of the *ACSL1* Gene Influence Milk Holstein Cows. Animals.

[24] Hill W.G., Robertson A. (1968). Linkage Disequilibrium in Finite Populations. Theor. Appl. Genet..

[25] Shi Y.Y., He L. (2005). SHEsis, a Powerful Software Platform for Analyses of Linkage Disequilibrium, Haplotype Construction, and Genetic Association at Polymorphism Loci. Cell Res..

[26] Browning B.L., Zhou Y., Browning S.R. (2018). A One-Penny Imputed Genome from Next-Generation Reference Panels. Am. J. Hum. Genet..

[27] Mao Y., Zhu X., Xing S., Zhang M., Zhang H., Wang X., Karrow N., Yang L., Yang Z. (2015). Polymorphisms in the Promoter Region of the Bovine Lactoferrin Gene Influence Milk Somatic Cell Score and Milk Production Traits in Chinese Holstein Cows. Res. Vet. Sci..

[28] Taylor J.F., Taylor K.H., Decker J.E. (2016). Holsteins Are the Genomic Selection Poster Cows. Proc. Natl. Acad. Sci. USA.

[29] Lee Y.M., Dang C.G., Alam M.Z., Kim Y.S., Cho K.H., Park K.D., Kim J.J. (2020). The Effectiveness of Genomic Selection for Milk Production Traits of Holstein Dairy Cattle. Asian Australas. J. Anim. Sci..

[30] KASAKOLU A., KONCAGÜL S. (2022). Effects of Different Methods and Genomic Relationship Matrices on Reliabilities of Genomic Selection in Dairy Cattle. Livest. Stud..

[31] Sharko F.S., Khatib A., Prokhortchouk E.B. (2022). Genomic Estimated Breeding Value of Milk Performance and Fertility Traits in the Russian Black-and-White Cattle Population. Acta Naturae.

[32] Kadri N.K., Guldbrandtsen B., Lund M.S., Sahana G. (2015). Genetic Dissection of Milk Yield Traits and Mastitis Resistance Quantitative Trait Loci on Chromosome 20 in Dairy Cattle. J. Dairy Sci..

