# Supplementary figures and images for: Association of NCOA6 Gene Polymorphism with Milk Production Traits in Chinese Holstein Cows
Source: Animals (Basel). 2025 May 19;15(10):1461. doi: 10.3390/ani15101461 (PMC12108186; doi:10.3390/ani15101461)

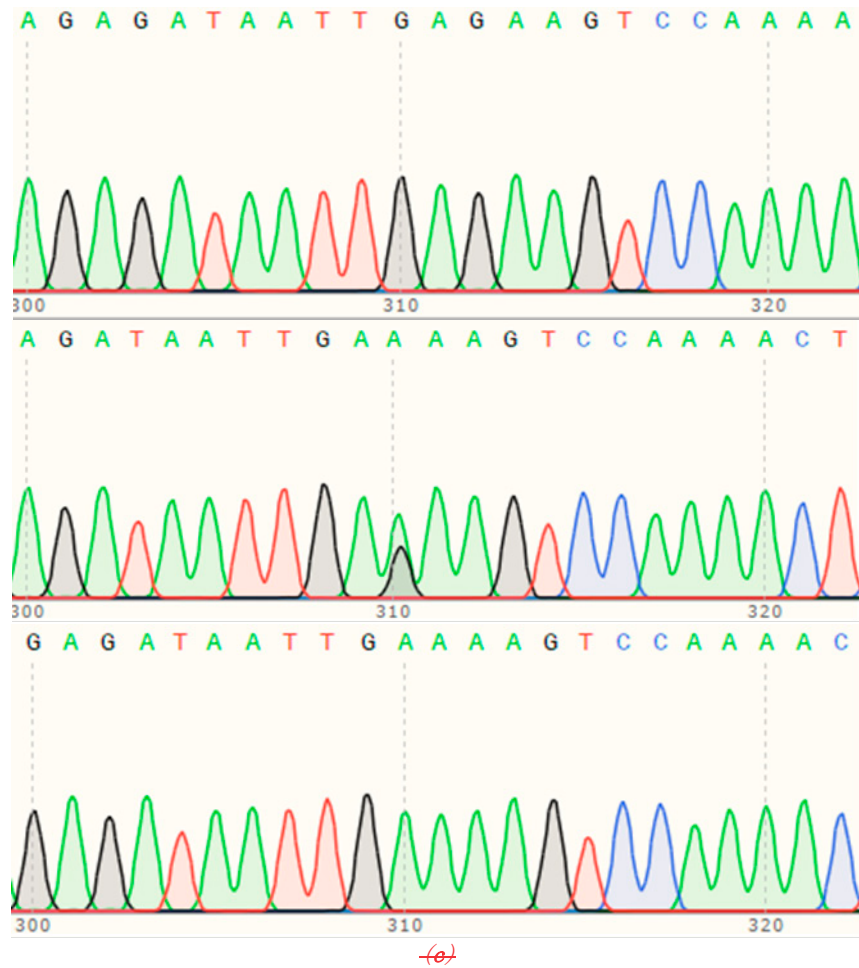

**Figure 1S.** Nuclear receptor coactivator 6 gene with the localization of one identified SNPs (NCOA6).

Supplement: Supplementary file 1 [file animals-15-01461-s001.zip › animals-3608549-supplementary.pdf]
